# Nanoparticle-Containing Hyaluronate Solution for Improved Lubrication of Orthopedic Ceramics

**DOI:** 10.3390/polym14173485

**Published:** 2022-08-25

**Authors:** Weihua Li, Yingying Wang, Wenwen Li, Lei Liu, Xiao Wang, Shiyong Song

**Affiliations:** 1 Orthopedics Department, Huaihe Hospital of Henan University, Kaifeng 475001, China; 2 School of Pharmacy, Henan University, Kaifeng 475004, China; 3 Henan Province Engineering Research Center of High Value Utilization to Natural Medical Resource in Yellow River Basin, Kaifeng 475004, China

**Keywords:** bovine serum albumin, sodium hyaluronate, biomimetic fluid, wear

## Abstract

Premature failure caused by inadequate lubrication of an artificial joint is a major problem. Inspired by engine lubrication, in which various additives are used to enforce the oil lubricant, here, a bench test of a biomimetic lubricating fluid containing different substances was carried out. Bovine serum albumin (BSA), in the form of both molecules and nanoparticles, was used as a functional additive. Compared with BSA molecules, BSA nanoparticles dispersed in HA solution served as more effective additives in the biomimetic lubrication fluid to minimize the friction and wear of ceramic orthopedic materials made of zirconium dioxide (ZrO_2_). Meanwhile, a tribo-acoustic study indicated that the “squeaking” problem associated with ZrO_2_ could be suppressed by the biomimetic fluid. Together with a cytotoxicity assessment, the BSA nanoparticle-incorporated biomimetic fluid was confirmed as a potential reagent for use in the clinic to maintain an even longer service life of artificial joints.

## 1. Introduction

Although remarkable achievements have been made in total hip arthroplasty (THA), the average effective life of artificial joints is not as long as expected by young patients who want to continue their work and live comfortably [1]. Second surgical repairs have to be performed upon the failure of the implants, which is associated not only with the painful suffering of patients but also with high costs, together with an extremely low success rate. There are several reasons for the failure of artificial joints. Among them, wear-induced periprosthetic osteolysis is the primary cause of hip implant failure [2,3]. Better control of the wear can be achieved by employing high-performance wear-resistant materials, such as ultrahigh molecular weight polymers, metal alloy, ceramic material, etc., for the fabrication of artificial implants [4]. Even so, implanted joints generally work under poor lubrication conditions, so little fluid is infiltrated from the surrounding tissue. With the increase in the popularity of ceramic friction pairs, clinical reports of squeaking in joint movements related to specific activities have been observed in recent years. The nature of squeaking is still not fully understood. Nevertheless, friction is typically considered the driving force in provoking sound emission [5,6,7]. Ceramic-on-ceramic (CoC) couples have outstanding wear resistance. If additional lubrication is provided, they will work for a longer time, together with a decreased occurrence of squeaking [8,9,10].

Ideal lubrication exists in human joints. The contact surfaces are covered by a layer of cartilage and, more importantly, are lubricated continuously by internally secreted synovial fluid (SF) [11]. SF is recognized as a biological super-lubricant; the friction coefficient is only 0.001–0.01, and for the human articular synovial membrane, it provides near-life-long low friction and low loss protection under the constantly changing load of the artificial joint due to motion [12]. Synovial fluid is an aqueous electrolyte solution whose main components include γ-globulin, phospholipids, hyaluronic acid, etc. [13,14]. The lubrication mechanism includes the fluid lubrication process, which also includes boundary lubrication or blending of the above two processes. Hyaluronic acid (HA) serves to thicken the lubrication fluid layer under fluid lubrication conditions [15]. In boundary lubrication, phospholipids and proteins may be adsorbed on the surface of articular cartilage. Artificial joints are so different from natural ones that a simple HA solution cannot provide adequate lubrication to artificial “rigid” joints. Even though natural and synthetic fluids have been formulated and studied for the lubrication of artificial joints, they have only been applied in wear tests of total joint replacements [16,17,18].

Similar to native SF with various functional components, the service behavior of the lubricating oil in construction machinery primarily depends on the performance of the additive components of the base oil. Since the 1990s, the effect of nano-additives as anti-wear additives of lubricating oil has been well known, and nanoparticles with unique optical, mechanical and tribological properties have received wide attention [19,20,21]. There are two types of views on the lubrication and anti-wear effects of nanoparticles. One is the micro-rolling of nanoparticles under the action of the friction force; another is that nanoparticles form a lubricating film deposited on the worn surface under the shear force [22]. A large number of studies have demonstrated that adding nanoparticles to lubricating oil can substantially enhance the lubrication performance and bearing capacity, with superior lubrication performance, self-repairing ability and excellent environmental friendliness [23,24]. Given the similarity between artificial joints and machine parts, the nanoparticle-assisted lubrication mechanism is also adaptable to artificial joints. Given the need for in vivo safety, artificial lubrication fluid should be formulated with biocompatible components that are both safe in vivo and effective in lubrication.

Certain amounts of adsorbed protein, including albumin and globulin, play an important role in the superlubricity of natural synovial fluid [14,25,26]. Furthermore, bovine serum albumin has good biocompatibility, and bovine serum albumin nanoparticles (BSA-NPs) have been widely used in new drug delivery systems [27,28,29,30]. Its targeting ability and biocompatibility in drug delivery systems have been confirmed by in vivo and in vitro experiments. In this study, a bionic lubricating fluid was formulated using HA and BSA-NPs. Their tribological properties were investigated as a lubricant for ZrO_2_ ceramic, which is one of the prosthesis materials. The rheological characteristics of the fluid were tested and correlated to their lubrication performance. Friction acoustics were also studied to assess the squeaking-reducing ability of the lubrication system. Finally, their biocompatibility was confirmed by the MTT assay.

## 2. Materials and Methods

### 2.1. Materials

Sodium Hyaluronate (HA, Mn = 39 K) was purchased from Huaxi Freda Biomedical Co., Ltd. (Jinan, China) Bovine serum albumin (BSA) and fetal bovine serum (FBS) were supplied by Gibco BRL (Gaithersburg, MD, USA). 3-(4,5-Dimethyl-2-thiazolyl)-2,5-diphenyl-2-H-tetrazolium bromide (MTT, 98%) was purchased from Solarbio Co., Ltd. (Beijing, China), and trypsin was provided by Invitrogen (Carlsbad, CA, USA). Tris hydrochloride (purity of 98%) was bought from Darui Fine Chemicals Co., Ltd. (Shanghai, China) and used to prepare the Tris buffer. Other solvents with analytical purity were purchased from Fuyu Fine Chemicals Co., Ltd. (Tianjin, China) and used without further purification.

### 2.2. Preparation of BSA Nanoparticle (BSA-NP)-Containing Lubrication Fluid

BSA nanoparticles were prepared following a previously reported procedure with slight modifications [31]. First, 60 mg of BSA powder was dissolved in 3 mL of Tris buffer solution (pH = 9), and then 8 mL of ethanol was added dropwise (about 1.5 mL/min) while stirring at a speed of 600 rpm until the solution changed from colorless to a blue opalescent colloidal solution. The solution was stirred for another 3 h at room temperature to remove ethanol. The mixture was transferred to a dialysis bag (Cut-off Molecular Weight: 8–14 k Da) and dialyzed exhaustively against deionized (DI) water for 36 h in order to remove residual ethanol. After lyophilization of the solution, a white powder of BSA-NPs was obtained. Morphological images were observed by transmission electron microscopy (TEM, JEM-100CX, Akishima, Japan). Dynamic laser scattering (DLS) measurement of particle size was carried out on a Zeta sizer Nano-ZS90 particle size/zeta potential analyzer (Malvern Instruments Inc, Malvern, UK). The stability of BSA-NPs was checked by measuring their sizes using DLS by dispersing the samples in DI water, PBS (pH = 7.4) and 50% FBS for 48 h at 37 °C.

Different lubrication fluids were formulated with different concentrations of HA and BSA-NPs; the three HA concentrations were: 5 mg/mL, 15 mg/mL and 30 mg/m, which were mixed with 5 mg/mL or 15 mg/mL BSA-NPs. For convenience, HA + BSA-NP (15 + 5) means that the mixture contains 15 mg/mL HA and 5 mg/mL BSA-NPs in DI water, HA + BSA-NP (15 + 15) means 15 mg/mL HA and 15 mg/mL BSA-NPs, and the other formulations hereafter follow the same scheme. As a control, BSA powder at the same concentrations as BSA-NPs was also formulated with HA lubrication fluids. Similarly, HA + BSA (15 + 15) means that the fluid was mixed with 15 mg/mL HA and 15 mg/mL BSA powders in DI water, and the other formulations follow the same scheme.

### 2.3. Tribological Tests

The lubrication properties of the fluids were tested utilizing a ball-on-plate method on a tribometer (UMT-2, CETR). Drops of fluids were added between a ZrO_2_ ball (diameter = 3 mm; average roughness: 20 nm) and a ZrO_2_ plate (20 mm × 4 mm; average roughness: 34 nm). The frictional coefficient was acquired under a normal force of 2 Newton. The motion frequency was set to 2 Hz, and the distance was 4 mm. Triple experiments were performed for each sample.

Wear tests were conducted on a four-ball tester (MRS-10A, SHIJIN Group Corp., Jinan, China). ZrO_2_ balls with a diameter of 12.7 mm (Karefonte Electronic S&T Co., Ltd., Shenzhen, China) were used. All balls were pre-cleaned with anhydrous ethanol and then ultrasonically cleaned for 30 min. The tests were conducted at room temperature (25 °C). A force of 50 N was applied to the lower balls, the rotational speed of the fourth ball was 1218 rpm, and the test time was 1800 s. A built-in optical microscope was employed to determine the wear scar diameter (WSD) of the three lower balls with an accuracy of 0.01 mm.

### 2.4. Rheological Measurements

The viscosity of the bionic joint fluid was measured on a rotational rheometer (Anton Paar MCR302, Graz, Austria) in the cone-and-plate (CP50-1, angle = 1.007°) mode at 25 °C temperature. During the experiment, the samples were dropped onto the lower plate and filled the gap between the cone and plates. The shear viscosity of the liquid was measured in the shear rate range (0.001–10s^−1^), and each sample was repeated 3 times. A rotational rheometer with a cone-and-plate mode of CP25–2/TG 1.999° cone angle was employed to study the linear and nonlinear viscoelastic properties of the samples. For the measurements, the gap distance between the plates was set constant at 100 μm. The temperature was held constant at 25 °C, and the test mode was “Frequency Sweep-Standard template, Amplitude Sweep-Standard template with LVE-Range analysis”. A frequency of ω = 0.063–500 rad/s was applied to the sample at a constant strain, γ = 0.5%, which was preliminarily determined to ensure a linear shear response. To examine the nonlinear behavior, a strain range of γ = 1–2000% was applied to the sample at constant ω = 6.3 rad/s, with vertical loading force F = 5 N, sliding speed V = 500 mm/s, and time T = 1200 s.

### 2.5. Tribo-Acoustic Testing

Tribo-acoustic testing was carried out on a home-built system reported by Hua et al. [7]. The system consists of an orthopedic biomaterial biotribometer, an acoustic acquisition system and a simu-anechoic chamber. Both tribological and acoustic data were acquired synchronously online. In the experiments, ZrO_2_ balls with a diameter of 12.7 mm were used as pins against ZrO_2_ disk samples, which were 20 mm in diameter and 4 mm in thickness, with surface roughness Ra between 0.5 and 1 μm. ZrO_2_ balls and ZrO_2_ disks were pre-cleaned with medical alcohol and then ultrasonically cleaned for 30 min. A force of 100 N was applied to the ZrO_2_ ball. The ball was rotated on the disk along a circular track of 10 mm diameter. The frequency was 1 Hz (the corresponding angular velocity of the disk was 6.28 rad/s), and the sliding distance was 31.4 mm/cycle with a constant sliding speed of 31.4 mm/s. Four types of lubricants, DI water, HA, HA + BSA and HA + BSA-NP, were compared. The HA (with a molecular weight of 390 kDa), BSA and BSA-NP content of the lubricant was 15 mg/mL. Each test lasted for 15 min at a temperature of 25 °C, and the relative humidity was 60 ± 10%. The sound pressure was acquired through an omnidirectional microphone placed 1 m away from the center of the tribometer, and then the signal was amplified and converted to digital data in Adobe Audition 3.0.1( Adobe Systems Inc., San Jose, CA, USA) The surface morphology of ZrO_2_ disk samples in the tribo-acoustic tests was examined using a Leica optical microscope.

### 2.6. In Vitro Cytotoxicity Study

The cytotoxicity of HA and HA + BSA-NP artificial joint lubricants was evaluated by MTT assay in Ana-1 cells (supplied by Shanghai Cell Bank of Chinese Academy of Sciences, Shanghai, China). The cells were seeded into a 96-well plate at a density of 4 × 10^3^ cells per well in 100 μL of RPMI-1640 media containing 10% FBS and incubated in a tissue culture incubator at 37 °C with 5% CO_2_ for 24 h. After 24 h, the medium of each well was removed, and then 100 μL of fresh complete medium containing various concentrations of HA lubricants or HA + BSA-NP artificial joint lubricants was added. The tests were carried out in four replicates for each concentration. The cells were further incubated for 48 h, the medium was aspirated, and 100 μL of 3-(4,5-dimethylthiazol-2-yl)-2,5-diphenyltetrazoliumbromide (MTT) solution (0.5 mg/mL) was added. The cells were incubated for 4 h, MTT solution was removed, and then 100 μL of DMSO was added to dissolve the purple formazan crystals. The optical densities at 570 nm were measured using a Universal Microplate Reader.

## 3. Results and Discussion

### 3.1. Preparation of the BSA-NP-Containing Biomimetic Synovial Fluid

**Figure 1:**
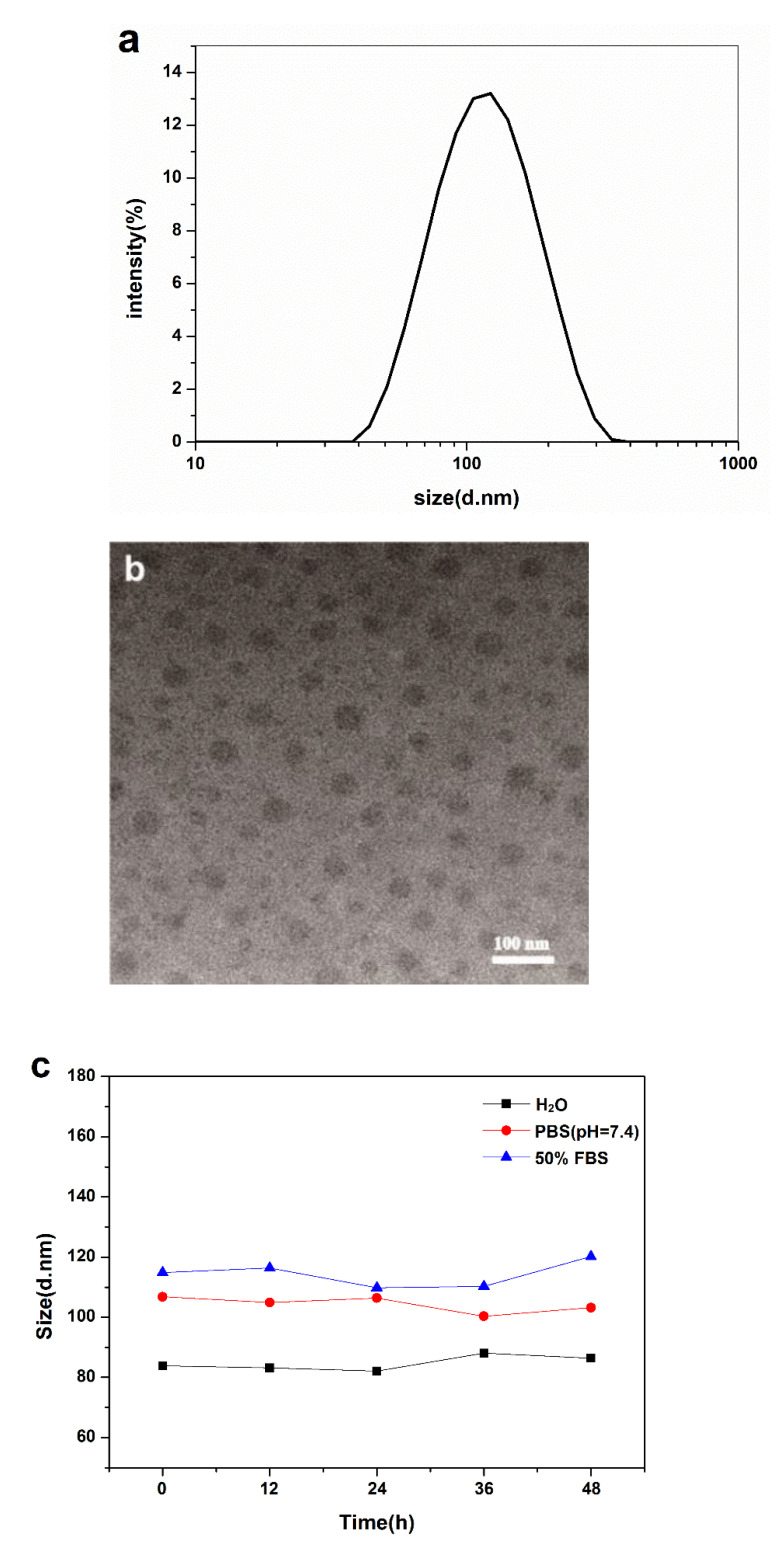
Characterization of BSA nanoparticles: size measurement by DLS (**a**); TEM image of BSA nanoparticles (**b**); particle size variation of BSA-NPs for 48 h in DI water, PBS (pH = 7.4) and 50% FBS (**c**).

The desolvation method is an efficient way to prepare BSA-NPs with a uniform size [8,9]. As shown in Figure 1a, the BSA-NPs had a diameter of 100 nm, as measured by dynamic light scattering (DLS), while it had a smaller size of around 60 nm in the TEM image (Figure 1b). From the TEM images, we can find that the nanoparticles have a regular spherical shape. The actual particle size is smaller than the corresponding hydrodynamic size measured by DLS. In addition, freeze-dried BSA-NP powder could be easily dispersed in DI water without any noticeable change in DLS size, resulting in a clear dispersion solution.

In order to ensure the colloid stability of BSA-NPs in a physiological environment, investigations of the size variation of BSA-NPs in DI water, PBS (pH = 7.4) and 50% FBS at 37 °C were performed. It was found that the measured sizes varied considerably in different mediums. This may be due to their different solution parameters, which are critical when performing DLS measurements. In 50% FBS, BSA-NPs had the largest DLS size, and in water, they were the smallest. The most important observation is that there was almost no change in nanoparticle size in each medium for as long as 48 h of incubation of BSA-NPs, which indicates that no aggregation of BSA-NPs occurred for 48 h. The stability of BSA-NPs could guarantee their long-term use as a bionic joint fluid additive.

BSA-NP-containing biomimetic fluids were formulated by mixing BSA-NPs with sodium hyaluronate (HA) in water. In healthy synovial fluid (SF), the molecular weight of HA is 6.3–6.7 M Da [1], with a concentration of about 2.5 mg/mL. In this work, the molecular weight of HA was 390 kDa. In order to maintain a similar viscosity to natural synovial fluid, the concentration of HA in this work should be rescaled according to the following formula [12]: κ × [HA] = 0.2~0.4 μM, where κ is equal to (MWHA/MWSF)^9/5^. According to the formula, the concentration of 390 kDa HA should range from 11.6 to 32.7 mg/mL.

**Figure 2:**
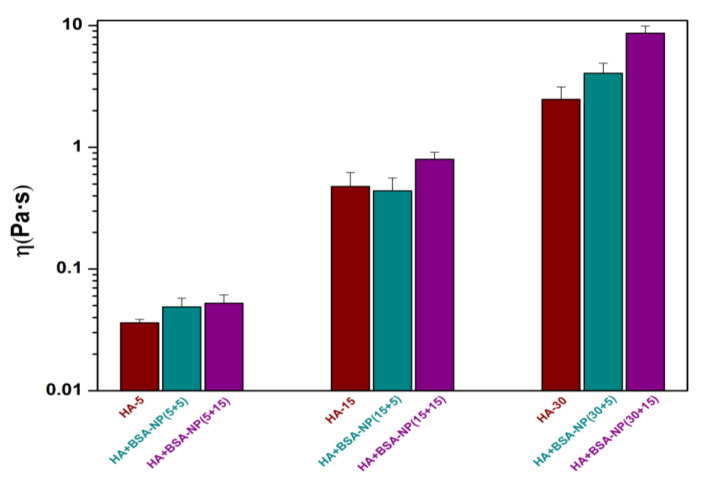
The viscosity of various fluids with different concentrations at a temperature of 25 °C (shear rate: 0.001–10 s^−1^): three concentrations (5 mg/mL, 15 mg/mL and 30 mg/mL) of HA were used and added with three different concentrations of BSA-NPs (0 mg/mL, 5 mg/mL and 15 mg/mL, respectively).

The viscosity of a fluid is closely related to its rheological and tribological properties. Therefore, studies on the shear viscosity of a fluid can explain the relationship of viscosity with fluid rheology and tribology to a certain extent. The viscosity of HA solutions with or without BSA-NPs was measured (Figure 2). The viscosity of HA solution increased with the concentration, and the addition of BSA-NPs could further increase the viscosity of HA solutions. There might exist intermolecular interactions between HA molecules and protein nanoparticles in the mixture. The BSA-NPs are wrapped in between HA macromolecules, which will increase the internal friction between the fluid molecules. To manifest the effect of nanoparticles, BSA powder was also formulated into HA solution to prepare a mixture of HA and BSA with final concentrations of 15 mg/mL and 15 mg/mL, respectively. The original viscosity of pure HA solution of 15 mg/mL (HA −15) was 0.477 Pa·s. The viscosity increased to 0.395 Pa·s and 0.800 Pa·s when adding the same amounts of BSA powder and freeze-dried BSA-NPs, respectively. Obviously, the addition of BSA-NPs was more effective in enhancing the viscosity in this case. This may be attributable to the formation of clusters by interactions between BSA-NPs and HA chains [12]. Generally, in a hydrodynamic lubrication regime, an increase in viscosity means an increase in friction. High viscosity is an important element in some circumstances. For example, synovial fluid in a healthy joint commonly has a higher viscosity than that of a diseased one.

### 3.2. Tribological Tests

**Figure 3:**
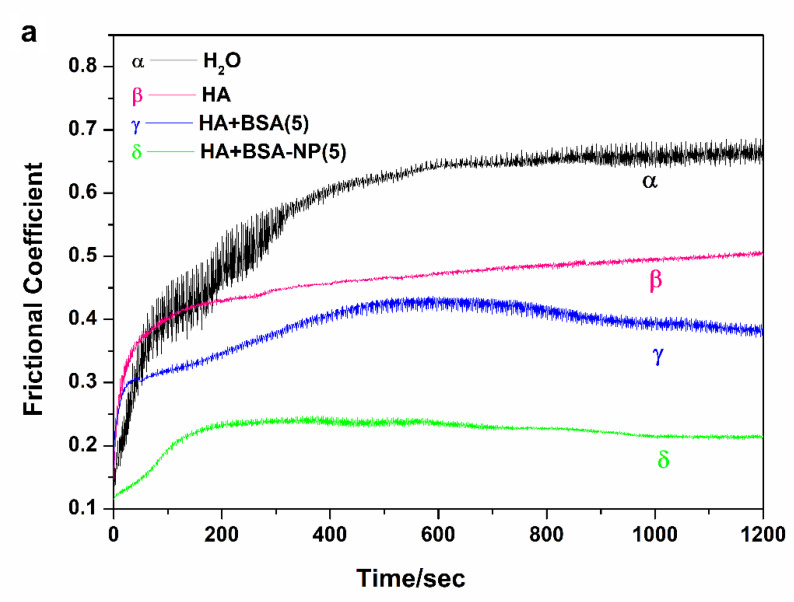

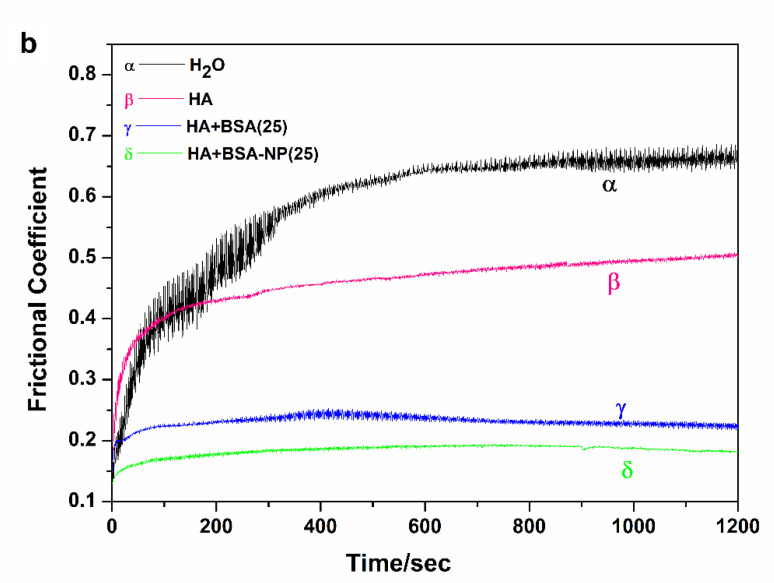
Frictional coefficient of H_2_O, HA and bionic synovial fluids containing different concentrations of BSA and BSA-NPs: (**a**) 5 wt% and (**b**) 25 wt%, under a load of 2 N, with the velocity equaling 8 mm/s, ZrO_2_-on-ZrO_2_.

The lubrication effect of the biomimetic fluid was assessed in ball-plate mode on a UMT-2 tribometer. Frictional coefficients were recorded when the ZrO_2_ ball rubbed against the ZrO_2_ plate under different lubricating conditions. HA solution with a concentration of 15 mg/mL was chosen as a basic solution. Different amounts of BSA or BSA-NPs were added to obtain final additive concentrations of 5 mg/mL (Figure 3a), 15 mg/mL (Figure 3b) and 50 mg/mL (Supplementary Material Figure S1). Water was used as a control to lubricate the ZrO_2_ counterparts and resulted in the highest coefficient of about 0.6 (Figure 3). The HA solution greatly reduced the friction by about 25%. The friction could be further decreased by adding BSA and BSA-NPs. Compared with BSA, BSA-NPs seemed more effective in reducing friction between the ceramic counterparts. This might be explained by the fact that BSA-NPs entrapped at the rubbing interface play a rolling lubrication role [32,33].

As for the BSA additive, when the concentration increased from 5 mg/mL to 15 mg/mL, the frictional coefficient dropped from about 0.4 to 0.2. With a further increase to 50 mg/mL, the decrease in friction was not as large. This indicates absorption saturation on the rubbing surfaces. Under the same conditions, the frictional coefficients changed little with the concentration of BSA-NPs, ranging from 5 mg/mL to 50 mg/mL, which indicates that only a small amount of BSA-NPs can play an effective role in reducing friction between ceramic parts.

From the above results, it was found that the BSA-NPs in the biomimetic fluid could reduce the friction of ceramic material at a load of 2 N. The effect of load on the friction coefficient was also tested under conditions of lubrication by HA solution (15 mg/mL) and BSA-NP-containing HA solution (15 mg/mL HA with 15 mg/mL BSA-NPs). It was discovered that loads ranging from 2 N to 10 N had almost no effect on frictional coefficients (Supplementary Material Figure S2). Besides the ZrO_2_-on-ZrO_2_ surfaces, the lubrication effect of the BSA-NP-containing fluids was further explored between a stainless steel ball and UHMWPE plate (SS-UHMWPE) (Supplementary Material Figure S3). With the same lubricants and a load of 5 N, the friction coefficients of SS-UHMWPE surfaces decreased by one order of magnitude relative to those of the ceramic surfaces. However, the trend in the friction coefficient reduction by the lubricants HA, HA + BSA and HA + BSA-NP was the same. Compared with the blank HA solution, after adding BSA-NPs, the friction coefficient was significantly lower than after adding BSA powders. Therefore, the impact of the BSA-NP additive on reducing the friction of HA solution is universal.

**Figure 4:**
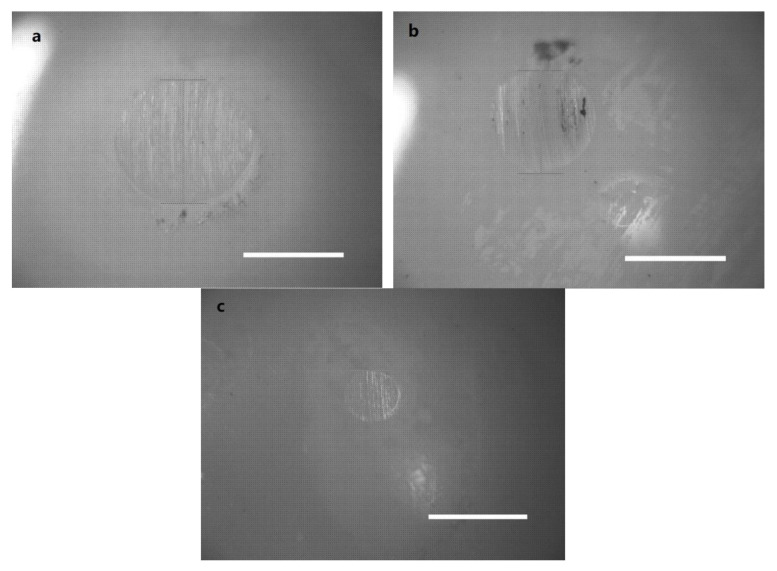
Images of the wear scar with HA (**a**); HA + BSA (**b**); HA + BSA-NPs (**c**). The concentration of HA was 15 mg/mL, and that of the additives was 15 mg/mL. The scale bar is 1 mm.

Under high load and relative motion, artificial joint movement is subjected to long-term friction, resulting in the severe wear of orthopedic materials, which is the leading cause of the loosening and failure of an artificial joint. In addition to the wear resistance of the orthopedic material itself, effective lubrication is also crucial to enhance the performance of the artificial joint. Herein, wear tests were conducted on a four-ball tester; the surface morphology of the wear scars is shown in Figure 4, and the diameter of the wear scars was measured by an optical microscope. According to the trajectory of the four balls, the shape of the wear scars was a regular circle. It was observed that the diameter of the wear scar on the ceramic balls lubricated by HA + BSA-NP fluid had the smallest value of 0.523 mm, indicating that it provided the most effective protection of the ZrO_2_ material.

Based on the tribology studies of the HA + BSA-NP biomimetic synovial fluid, the following conclusions can be drawn: the addition of the BSA-NP additive significantly reduces the friction coefficient of HA solution, and combined with the addition of the BSA-NP additive, an enhancement of the viscosity of HA solution can be found; if the viscosity of the bionic joint fluid is higher, the friction coefficient is smaller, and they may be negatively correlated. We suspected that the outstanding friction-reducing effect of BSA-NPs may be due to the formation of an HA-NP complex consisting of BSA-NPs and HA macromolecules; a stable lubrication film is formed at the interface of the friction pair, which has a good lubrication effect.

### 3.3. Rheological Measurements

**Figure 5:**
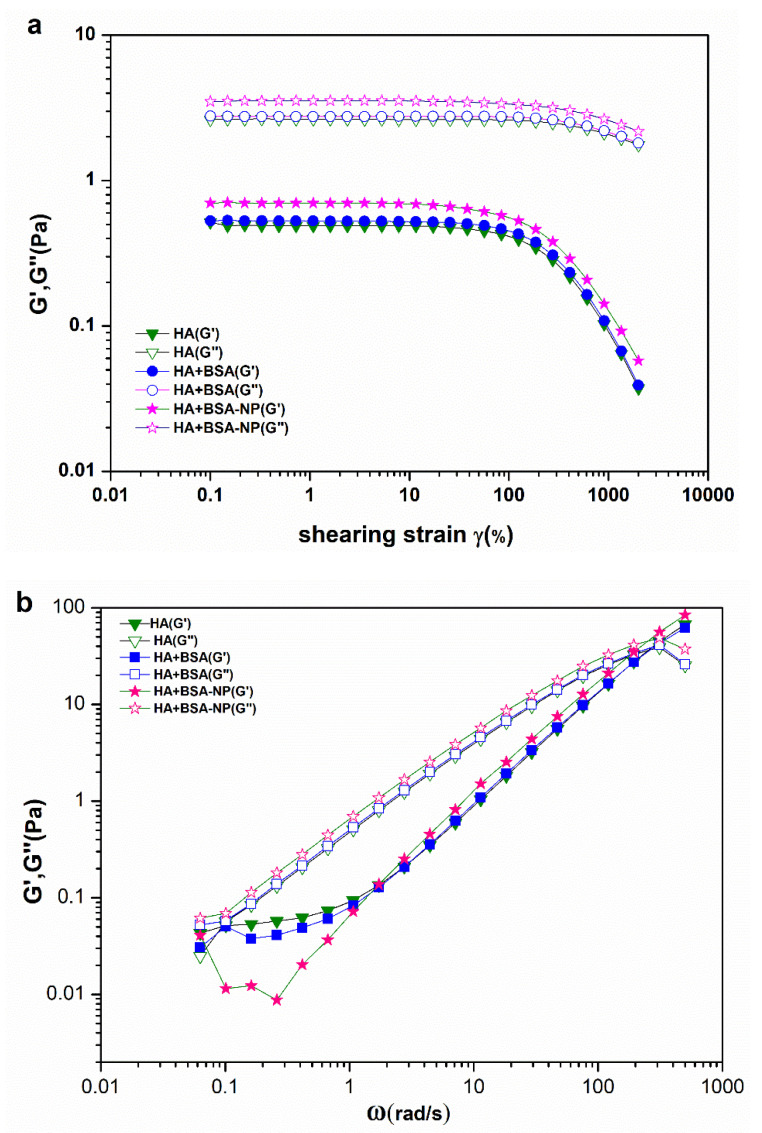
The elastic modulus (G′) and viscous modulus (G″) of HA (15 mg/mL) with or without BSA-NPs and BSA (15 mg/mL): strain scanning ranging from 0.1% to 2000% at a frequency of 6.3 rad/s (**a**); frequency scanning ranging from 0.063 rad/s to 500 rad/s at a strain of 0.5% (**b**).

The viscoelastic modulus is an important parameter in fluid mechanics, which is closely related to the friction reduction and anti-wear performance of biomimetic fluids. Strain scanning was performed to record the variation in moduli, including the elastic modus (G′) and viscous modus (G″), along with the change in strain (γ) (Figure 5a). For the three fluids, the viscosity modulus G″ was definitely higher than the elastic modulus G′, indicating a predominantly viscous fluid with or without added BSA and BSA-NPs. When γ < 100%, all solutions were in the linear viscoelastic region. When γ rose beyond 100%, the fluid entered the nonlinear viscoelastic region, and the curve displayed an exponential decreasing tendency. Evidently, HA solution mixed with BSA-NPs gained an increase in both G″ and G′ values. However, there was no change in HA solution when adding BSA.

The linear viscoelastic properties of HA solution and its mixtures are shown in Figure 5b. The angular frequency (ω)-dependent elastic modulus (G′) and viscous modulus (G″) were measured at a constant strain (γ = 0.5%), at which a linear response is ensured. All three samples exhibited typical viscoelastic behavior of a Maxwellian fluid characterized by a crossover frequency in the linear shear spectra. When ω < 300 rad/s, G″ was greater than G′, demonstrating the predominantly viscous fluid of HA aqueous suspension solution for both cases with and without added BSA and BSA-NPs. When ω > 300 rad/s, G′ showed an upward trend, while G″ showed an exponential declining trend, and G′ became dominant.

The modulus test indicated that when BSA-NPs were added to HA, the -OH and -COOH of amino acids in protein nanoparticles interacted with HA molecules through hydrogen bonds to form HA–nanoparticle complexes, strengthening the HA intermolecular entanglement, and the elastic network structure of HA became more solid. The rheological properties changed with the enhancement of the mechanical strength of the fluid lubrication film, which would improve the tolerance to high load and ensure the long-term lubrication effect of the bionic joint liquid.

### 3.4. Tribo-Acoustic Study

**Figure 6:**
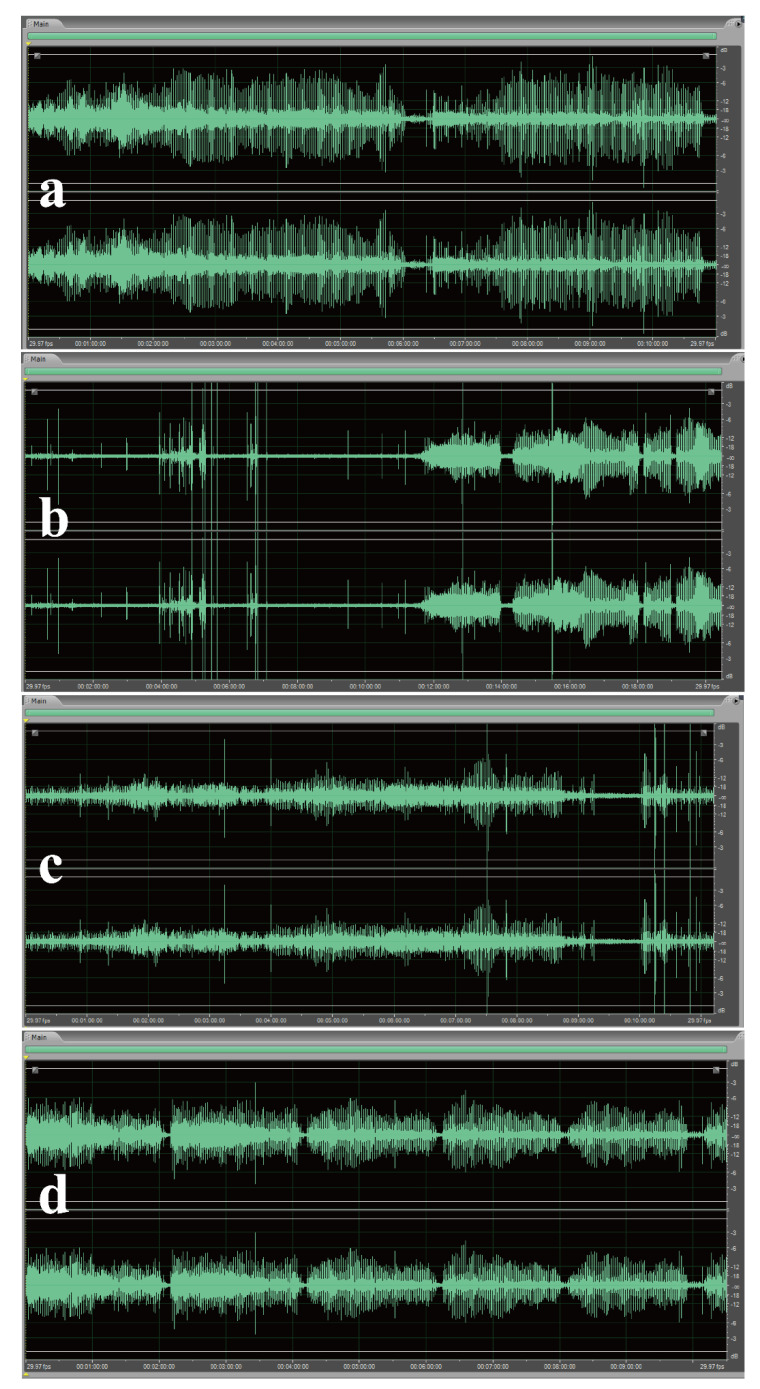
Sound pressure signal in a tribo-acoustic test with different lubricants: DI water (**a**); HA (**b**); HA + BSA (**c**); HA + BSA-NP (**d**). The concentration of HA was 15 mg/mL, and that of the additives was 15 mg/mL (test conditions: ZrO_2_ ball-on-plate, a load of 100 N and a frequency of 1 Hz).

The problem of joint “squeaking” is a serious problem in ZrO_2_ ceramic materials compared with other kinds of artificial joint materials [5,6,34]. The squeaking is not always painful, but it causes an unsatisfying feeling for patients. Various factors have been found to be the origin of squeaking, among which friction is the most important one [35,36]. From this point of view, adequate lubrication can decrease the incidence of ceramic-on-ceramic squeaking. Figure 6 shows the sound pressure acquired when the ZrO_2_ ball rubbed against the ZrO_2_ plate during lubrication with DI water, HA solution and HA solution with BSA and BSA-NPs. The sound pressure signal of DI water showed a cycle of amplifying, falling, increasing and falling; the whole process was unstable, and the pressure range was −3~+3 dB (Figure 6a). For pure HA solution, the average amplitude fell into a narrower range between −12~+12 dB, but intermittent sharp sounds sometimes appeared (Figure 6b). Even when BSA was included, the sound pressure was still not very steady, and ultrahigh sound pressure frequently appeared. Comparably, the BSA-NP-containing HA solution generated the most stable sound pressure, which indicates that fewer squeaking events were produced (Figure 6d). Therefore, among the three lubricating fluids, HA + BSA-NPs can decrease the “squeaking” of the ZrO_2_ ceramic joint in terms of both its power strength and occurrence rate.

**Figure 7:**
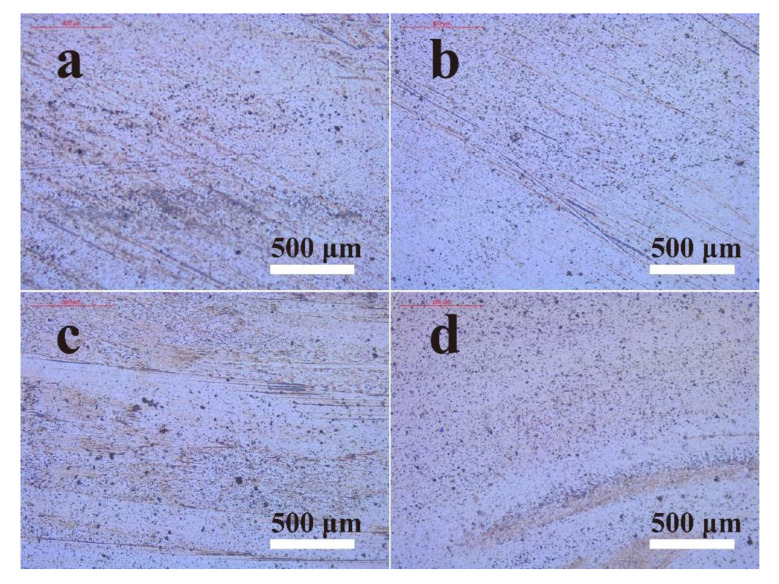
Optical images of the ZrO_2_ disk surface after a tribo-acoustic test with various lubricants: DI water (**a**); HA solution (**b**); HA + BSA solution (**c**); HA + BSA-NP solution (**d**).

As for the morphology of the disk wear surfaces, obvious traces of wear and carbonized wear debris were found when lubrication was provided by DI water, HA and HA + BSA (Figure 7). Conversely, wear surfaces lubricated in HA + BSA-NP fluid appeared to be polished and much smoother, and abrasion was less obvious with no noticeable wear debris.

### 3.5. In Vitro Cytotoxicity Study

**Figure 8:**
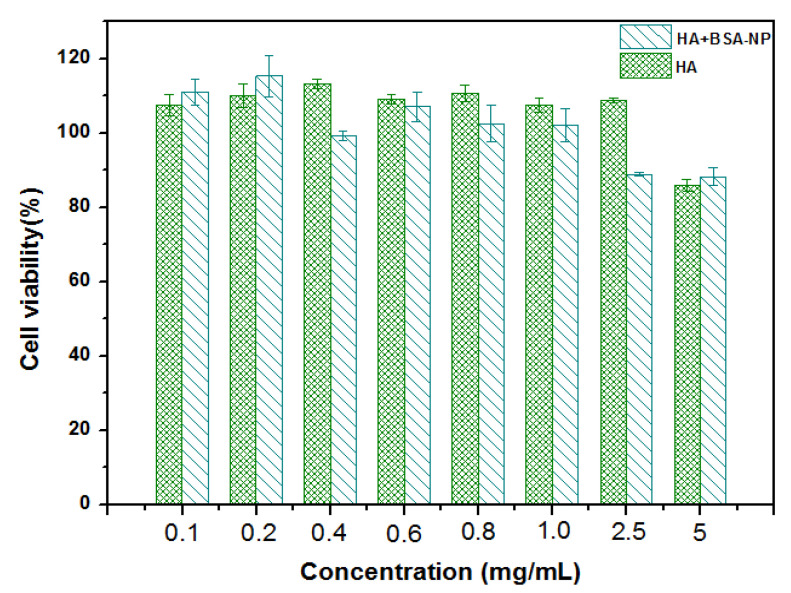
Cell viability of Ana-1 cells exposed to HA and HA + BSA-NPs.

The toxicity of the bionic joint lubricants HA and HA + BSA-NPs were tested in Ana-1 cells using the MTT assay. It was concluded that both HA and HA + BSA-NPs were non-toxic and biocompatible up to a concentration of 5.0 mg/mL (Figure 8). The cell viability was above 90% after incubation with HA and HA + BSA-NPs for 48 h. The results showed that the formulated biomimetic liquid was biocompatible and applicable for artificial joint lubrication.

## 4. Conclusions

In this preliminary study, a biomimetic fluid was successfully formulated based on a BSA nanoparticle-containing hyaluronate solution. The effective lubrication performance of the fluid was confirmed for ZrO_2_ ceramic orthopedic materials. Compared to pure HA and BSA molecule-containing HA solutions, the incorporation of BSA in the form of nanoparticles was essential for the friction and wear reduction of ZrO_2_ material. In addition, viscosity measurements were carried out to show that the addition of BSA-NPs can markedly improve the viscosity of HA solution. A rheological study revealed that the formed complex between HA and nanoparticles is likely the reason for the improved tribological performance. Furthermore, a tribo-acoustic study showed that the nanoparticle-based biomimetic fluid paves the way to reducing the incidence of the “squeaking” phenomenon in ZrO_2_ ceramic implants. An in vitro cytotoxicity study showed that the HA + BSA-NP biomimetic fluid has excellent biocompatibility. Even though there is a long distance from bench to bedside, this work provides a potential way to prolong the service life of artificial joints by reducing their wear.

## Data Availability

The data presented in this study are available on request from the corresponding author.

## Supplementary Materials

The following supporting information can be downloaded at: https://www.mdpi.com/article/10.3390/polym14173485/s1. Figure S1: Frictional coefficient of H_2_O, HA and HA solution containing 50 mg/mL BSA and BSA-NP under load of 2N, velocity equaling 8 mm/s ZrO_2_-on -ZrO_2_; Figure S2: Evolution of Frictional coefficient with time under different loads at constant velocity of 8 mm/s and lubricated by HA solution (15 mg/mL) with or without BSA-NPs (15 mg/mL); Figure S3: Evolution of Frictional coefficient between SS-UHMWPE with sliding distance under lubrication of HA solution (15 mg/mL) with or without additive (15 mg/mL) and the load is 5N, at sliding rate equaling 0.5 m/s.
