# Supplementary material for: Nanoparticle-Containing Hyaluronate Solution for Improved Lubrication of Orthopedic Ceramics
Source: Polymers (Basel). 2022 Aug 25;14(17):3485. doi: 10.3390/polym14173485 (PMC9460720; doi:10.3390/polym14173485)
Supplement: Supplementary file 1 [file polymers-14-03485-s001.zip › polymers-1870059-supplementary.pdf]

Supplementary Materials

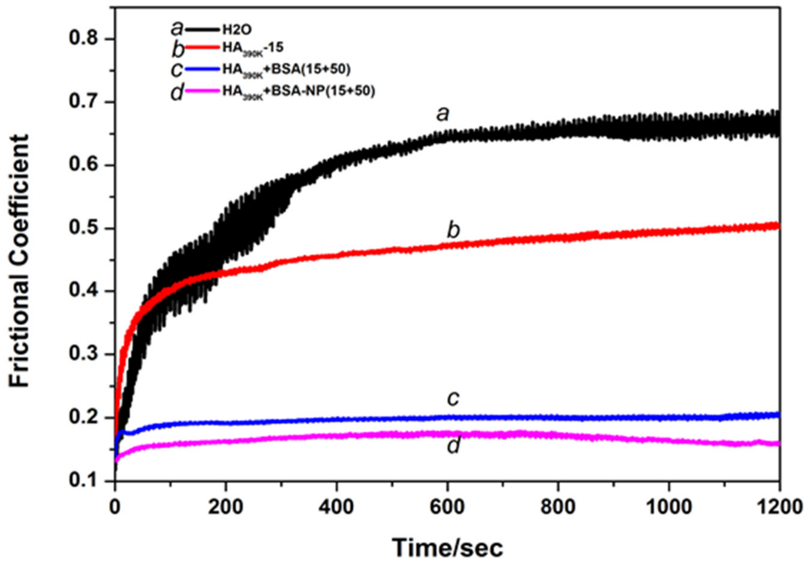

Figure S1. Frictional coefficient of H_2O, HA and HA solution containing 50mg/mL BSA and BSA-NP under load of 2N, velocity equaling 8mm/s ZrO_2-on -ZrO2.

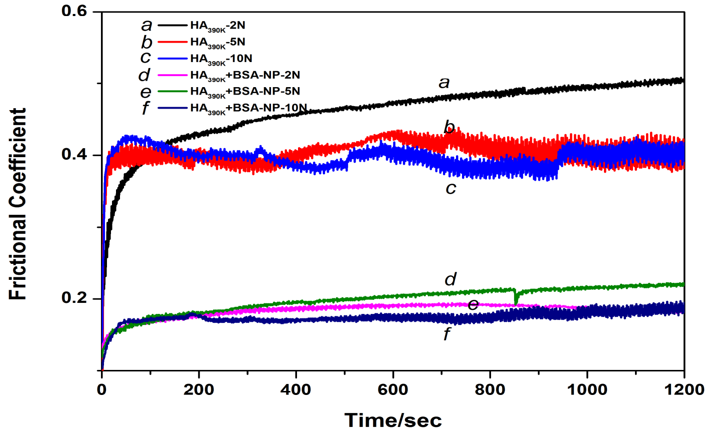

Figure S2. Evolution of Frictional coefficient with time under different loads at constant velocity of 8mm/s and lubricated by HA solution(15mg/mL) with or without BSA-NPs (15mg/mL).

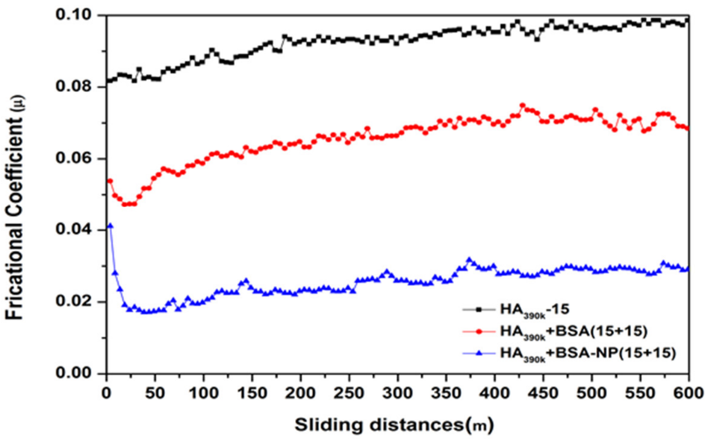

Figure S3. Evolution of Frictional coefficient between SS-UHMWPE with sliding distance under lubrication of HA solution(15mg/mL) with or without additive (15mg/mL) and the load is 5N, at sliding rate equaling 0.5m/s.
